# Therapeutic Potential of Heme Oxygenase-1 in Aneurysmal Diseases

**DOI:** 10.3390/antiox9111150

**Published:** 2020-11-19

**Authors:** Wei-Cheng Jiang, Chen-Mei Chen, Candra D. Hamdin, Alexander N. Orekhov, Igor A. Sobenin, Matthew D. Layne, Shaw-Fang Yet

**Affiliations:** 1 Institute of Cellular and System Medicine, National Health Research Institutes, Zhunan 35053, Taiwan; wcjiang@nhri.edu.tw (W.-C.J.); 060714@nhri.edu.tw (C.-M.C.); cdh@nhri.edu.tw (C.D.H.); 2 Molecular and Biomedical Sciences, Department of Life Sciences, National Central University, Zhongli District, Taoyuan City 32001, Taiwan; 3 Institute of Human Morphology, 3 Tsyurupa Street, 117418 Moscow, Russia; a.h.opexob@gmail.com; 4 Laboratory of Medical Genetics, National Medical Research Center of Cardiology, 15A 3-rd Cherepkovskaya Street, 121552 Moscow, Russia; igor.sobenin@gmail.com; 5 Department of Biochemistry, Boston University School of Medicine, Boston, MA 02118, USA; mlayne@bu.edu

**Keywords:** abdominal aortic aneurysm, intracranial aneurysm, subarachnoid hemorrhage, heme oxygenase-1, inflammation, oxidative stress

## Abstract

Abdominal aortic aneurysm (AAA) and intracranial aneurysm (IA) are serious arterial diseases in the aorta and brain, respectively. AAA and IA are associated with old age in males and females, respectively, and if rupture occurs, they carry high morbidity and mortality. Aneurysmal subarachnoid hemorrhage (SAH) due to IA rupture has a high rate of complication and fatality. Despite these severe clinical outcomes, preventing or treating these devastating diseases remains an unmet medical need. Inflammation and oxidative stress are shared pathologies of these vascular diseases. Therefore, therapeutic strategies have focused on reducing inflammation and reactive oxygen species levels. Interestingly, in response to cellular stress, the inducible heme oxygenase-1 (HO-1) is highly upregulated and protects against tissue injury. HO-1 degrades the prooxidant heme and generates molecules with antioxidative and anti-inflammatory properties, resulting in decreased oxidative stress and inflammation. Therefore, increasing HO-1 activity is an attractive option for therapy. Several HO-1 inducers have been identified and tested in animal models for preventing or alleviating AAA, IA, and SAH. However, clinical trials have shown conflicting results. Further research and the development of highly selective HO-1 regulators may be needed to prevent the initiation and progression of AAA, IA, or SAH.

## 1. Introduction

### 1.1. Aneurysmal Diseases

Cardiovascular diseases remain the leading cause of death worldwide, particularly in developed countries. Of all cardiovascular diseases, aneurysmal diseases are not as well-known or studied as coronary heart diseases. An aneurysm is a bulge or ballooning in a blood vessel and the underlying problem in aneurysmal disease is weakening of the arterial wall leading to progressive dilatation and risk of rupture [1]. Aneurysms are classified by their location in the vasculature: abdominal aortic aneurysms (AAAs) occur in the abdominal aorta and intracranial aneurysms (IAs) develop in the cerebral arteries. The global prevalence rate of AAA in 2010 ranged from 7.88 to 1229 per 100,000 individuals in the 40–44 and 65–69 age groups, respectively [2]. The overall death rate of AAA increased from 2.49 to 2.78 per 100,000 individuals from 1990 to 2010 [3]. Men with advanced age are more prone to develop AAAs and male gender is also associated with a four-fold higher risk of AAA [3,4,5]. With the global average increase in age and lifespan [6], the age-standardized mortality of AAA has also increased, indicating a concerning rise in total AAA incidence [1,3,7].

Prospective autopsy and angiographic studies reveal that 3.6–6% of the population has an IA, which is primarily localized in proximal arterial bifurcations in the circle of Willis [8]. The estimated lifetime medical costs, including hospitalization and surgery, for patients with unruptured IAs in the United States is over USD 500 million [9]. Although AAAs and IAs are both arterial aneurysms, IAs are characterized by a distinct saccular morphology that is different from AAAs. In contrast to the male prevalence of AAAs, IAs have higher prevalence in females [10,11] and increase rapidly with age [12]. Computed tomography (CT) and magnetic resonance imaging (MRI) are progressively more common in clinical practice, resulting in the more frequent detection of unruptured IAs. Therefore, there is an increasing need for pharmacological treatments to suppress IA progression and rupture.

Subarachnoid hemorrhage (SAH) is a neurological emergency characterized by bleeding into the space surrounding the brain that is filled with cerebrospinal fluid. SAH without a preceding trauma is caused by the rupture of an IA in 80% of cases, accounting for 5–10% of all strokes in the United States [13,14]. The multifactorial pathophysiology following SAH often results in irreversible brain damage. The amount of subarachnoid blood detected on the initial CT of patients with aneurysmal SAH has independent predictive power for the occurrence of delayed cerebral ischemia [15]. SAH is also associated with a higher rate of medical complications and 40–50% mortality [16]. SAH affects younger individuals than those affected by other forms of stroke [13]. Despite only accounting for 3% of all strokes each year [17], SAH stroke is a major cause of premature mortality, accounting for 27% of all stroke-related years of potential life lost before the age of 65 [18]. In addition, the survivors often experience long-term functional disability and/or cognitive impairment [19]. Aneurysmal SAH remains an overwhelmingly destructive form of stroke and contributes to a substantial medical and economic burden, with a magnitude similar to that of ischemic stroke [20]. The estimated annual cost of SAH in the United States is USD 1.755 billion, more than three-fold higher than the costs associated with unruptured IAs [9]. Factors associated with an increased risk of aneurysm rupture include race, hypertension, cigarettes and alcohol use, and IA diameter larger than 7 mm [21,22].

### 1.2. Current Treatments for Aneurysmal Diseases

AAAs often grow slowly and are usually asymptomatic, making them difficult to detect. Patients with abdominal or back pain are recommended to have ultrasound imaging to determine whether a AAA is present [23]. Although the risk of rupture for small AAAs (3.5–5.5 cm diameter) is low [24], large AAAs (>5.5 cm diameter) are prone to rupture, resulting in a mortality rate of 80–85% [4]. Several approaches are proposed to attenuate the progression of small AAAs including the management of atherosclerotic risk factor factors—hypertension, hyperlipidemia, and diabetes. Furthermore, AAAs should be monitored regularly [23]. For large or symptomatic AAAs, prophylactic operative repair by open surgery or endovascular repair is necessary [23,25]. Patients with ruptured AAAs that present with severe abdominal or back pain, hypotension, and palpable abdominal mass need immediate interventional procedures. Although open repair is the treatment of choice for most ruptured AAAs, endovascular stent-graft repair is warranted in many cases because it is less invasive and has a lower morbidity and mortality [26].

Treatment options for IAs are limited to invasive therapies including surgical clipping and endovascular embolization. Randomized, controlled trials suggest that IAs that are judged to be treatable by surgical interventions are best treated by endovascular procedures, although open surgery maybe preferred in certain cases [13]. Data from the International Study of Unruptured Intracranial Aneurysms reported non-negligible risk of these procedures, with combined mortality and morbidity at 1 year of 12.6% and 9.5%, respectively [27]. Currently, endovascular coiling to block blood flow is often preferred for elderly patients because it is minimally invasive and has lower associated mortality and morbidity [27]. However, endovascular coil embolization has a high rate of recurrence and needs retreatment [28]. To prevent aneurysmal SAH, proper management of risk factors similar to those of AAAs including controlling hypertension may prevent IA rupture. Nevertheless, for ruptured IAs, surgical clipping or endovascular coiling should be performed as early as feasible in the majority of patients to reduce the rate of bleeding after aneurysmal SAH. For patients judged to be technically amenable to both interventions, endovascular coiling should be considered [29].

### 1.3. Opportunity of Heme Oxygenase-1 in Aneurysmal Diseases

Surgical interventions are required to repair large AAAs and IAs. For small unruptured aneurysms, pharmacological agents that could prevent or even regress these aneurysms would alleviate the need for patients to undergo major surgical procedures [30,31]. Despite that, there are some differences in the etiology between AAAs and IAs; these vascular diseases share similar risk factors, including age, smoking, hypertension, and family predisposition [11]. Furthermore, many pathogenic mechanisms and characteristics of AAA have been reported in IAs, including oxidative stress, inflammation, endothelial dysfunctions, phenotypic modulation and loss of vascular smooth muscle cells (VSMCs), leukocytes infiltration, and vascular remodeling. Elastin fragmentation and degradation of extracellular matrix (ECM) by matrix metalloproteinases (MMPs) result in arterial wall weakening [32]. Given that both inflammation and oxidative stress contribute to AAAs and IAs, pharmacological therapeutic strategies aimed at reducing inflammation and reactive oxygen species (ROS) levels appear to be promising approaches for preventing/treating these devastating vascular diseases.

**Figure 1:**
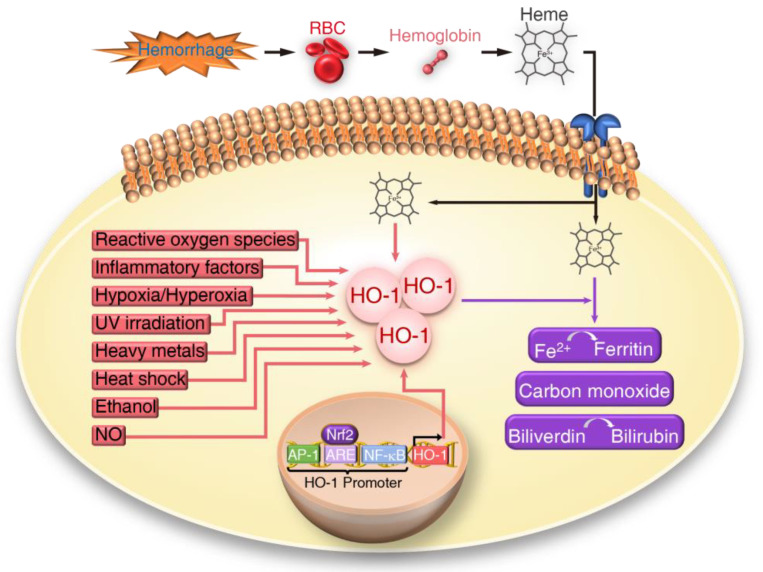
Schema of heme oxygenase-1 (HO-1) upregulation. In response to cellular stress, HO-1 can be strongly induced by many factors, such as reactive oxygen species, inflammatory factors, hypoxia/hyperoxia, UV irradiation, heavy metals, heat shock, ethanol, heme, and nitric oxide (NO). Heme is not only a substrate for HO-1 but also a stimulus for HO-1 upregulation. A source of heme is from breakdown of hemoglobin released from lysed blood due to hemorrhage. Factors that upregulate HO-1 also increase Nrf2 expression, which then binds to antioxidant response elements (ARE) in the HO-1 promoter to induce HO-1 gene transcription. Other cis-elements on the HO-1 promoter include AP-1 and NFκB sites contribute to transcriptional regulation of HO-1. After induction, HO-1 catalyzes the oxidation of heme to generate biologically active molecules, including carbon monoxide (CO) that has antioxidant and anti-inflammation functions. Ferrous iron (Fe^2+^) can induce ferritin expression for iron sequestration. Additionally, biliverdin is subsequently reduced to bilirubin by biliverdin reductase which is an additional antioxidant.

Heme oxygenase (HO) is an endoplasmic reticulum-associated enzyme that catalyzes the degradation of the prooxidant heme and generates carbon monoxide (CO), biliverdin, and ferrous iron [33,34,35]. Ferrous iron can induce ferritin expression to sequester iron while bilirubin, converted from biliverdin, is an antioxidant. In addition to its anti-inflammatory functions, recent studies indicate that bilirubin also promotes health via hepatic pathways [36]. CO has both anti-inflammatory and antioxidant properties. Two major HO isoforms have been identified (HO-1 and HO-2). HO-2 is constitutively expressed; however, the inducible HO-1 isoform is most extensively studied. Numerous studies have revealed that HO-1 is normally expressed at low levels in most tissues; in response to pathological stress, HO-1 is significantly induced [37], probably as an endogenous mechanism to protect against tissue injury (Figure 1). Various studies have demonstrated the protective effects of HO-1 in cardiovascular health. In particular, in an HO-1-deficient human, excessive vascular inflammation has been observed [38,39]. In recent years, naturally derived HO-1 inducer has been employed as an approach to treating exacerbated inflammation [40]. Based on the properties of HO-1, induction of HO-1 offers a unique opportunity as a therapeutic target for aneurysmal disorders.

## 2. HO-1 in Abdominal Aortic Aneurysm

AAA is a localized dilatation of the abdominal aorta exceeding the normal diameter by more than 50% [41]. The unique hemodynamic environment of the infrarenal aortic region predisposes it to site-specific degenerative changes. The infrarenal aorta is the most common site of aortic aneurysm formation. Differential hemodynamic factors and regional features may explain the specific anatomic localization of AAA [42]. AAA is a chronic vascular disease caused by progressive inflammation and oxidative stress-induced injury, resulting in permanent aortic segmental dilatation, which may progress to dissection or rupture [43,44]. In addition to inflammatory cell infiltration and ROS production, AAA is also associated with depletion of medial VSMCs and degradation or remodeling of aortic wall ECM [45]. Old age, male gender, smoking history, high blood pressure, and hereditary predisposition are the most critical contributing factors to AAA [5]. In addition to the common cardiovascular risk factors, increasing evidence indicates separate pathogenic mechanisms underly the development of both AAA and arterial occlusive diseases [46,47]. AAA is traditionally detected by ultrasound; however, prognostic markers will help identify patients with AAA. A prospective study has identified several circulating biomarkers (white blood cell count, fibrinogen, D-dimer, troponin T, N-terminal probrain natriuretic peptide, and high-sensitivity C-reactive protein) that are strongly associated with AAA incidence [48].

Other than environmental (common cardiovascular) risk factors, genetic risk factors also contribute to the development of AAA. Meta-analysis of genome-wide association studies identified 10 disease-specific risk loci, including MMP-9, IL6R, LDLR, ERG, and SMYD2. These risk loci do not always associate with coronary artery disease, blood pressure, lipids, or diabetes mellitus [49], which emphasizes that distinct mechanisms underly the pathogenesis of AAA vs. other cardiovascular diseases. A recent genome-wide association study in the Million Veteran Program identified 14 more novel AAA risk loci, including LDAH and ADAMTS8 [50]. Although not identified within these 24 risk loci, the stress response gene HO-1 has been shown to be involved in AAA development and progression. The regulation of the HO-1 expression is controlled by polymorphisms in the length of GT repeats in the proximal promoter, and shorter GT repeats (<25 GT) exhibit greater HO-1 upregulation and anti-inflammatory responses compared with longer repeats [51]. Intriguingly, patients with AAA are less frequently carriers of short (<25 GT) repeats in the HO-1 gene promoter than patients with atherosclerosis or healthy subjects, suggesting short GT repeats facilitate upregulation of HO-1 and serve a protective function against the development of AAA [52].

Genetic mutations also contribute to the development of aortic aneurysm. For example, Marfan syndrome is an inherited autosomal dominant disorder of connective tissue, affecting the cardiovascular system [53] and has an incidence of approximately 1 in 9800 in England [54]. Marfan syndrome is often caused by mutations in the fibrillin-1 gene, resulting in abnormalities of fibrillin metabolism and altered elastic microfibril and ECM assembly in the aorta in most patients [55]. Progressive dilatation of the aorta with subsequent dissection or rupture is the main cause of mortality. In addition to fibrillin-1 mutations, transforming growth factor β receptor 2 (TGFBR2) and TGFBR1 also cause Marfan syndrome and these pathways regulate ECM synthesis and homeostasis [56,57,58]. Elastic fiber fragmentation, paucity of VSMCs, deposition of collagen, and mucopolysaccharide between the cells of the medial layer are found in both Marfan syndrome patients and those with AAA. Thus, therapeutic strategies may relieve symptoms of AAA and Marfan syndrome.

Although prophylactic operative repair is the only option for of larger aneurysms, developing pharmacological therapies to attenuate or stop AAA progression, and therefore avoiding the need for surgical repair, still has great potential [59]. Therein, HO-1 acts as a stress response protein and plays an important cytoprotective role against injury with its antioxidative and anti-inflammatory properties in cardiovascular disease [35,60]. Accordingly, various studies have investigated the influence of HO-1 on AAA. From a biofluiddynamics perspective, sustained shear stress dramatically increased HO-1 expression and inhibited elevation of ROS and enlargement of the aortic wall in a rat pancreatic elastase-induced AAA model [61], implicating a protective function of HO-1 in AAA formation. Haploinsufficient HO-1 in mice (one allele of HO-1 gene is mutated or deleted) were more prone to elastase-induced AAA than wild type mice and exhibited higher levels of macrophage recruitment and inflammatory cytokines in the blood [62]. Further supporting a critical role of HO-1 in AAA development, we demonstrated unequivocally that HO-1 expression was crucial in AAA growth and severity. We showed that in the angiotensin II-induced AAA model, complete loss of HO-1 exacerbated AAA incidence and rupture rate in mice, concomitant with increased ROS level, VSMC apoptosis, severe elastin degradation, macrophage infiltration, and MMP activation [63].

**Figure 2:**
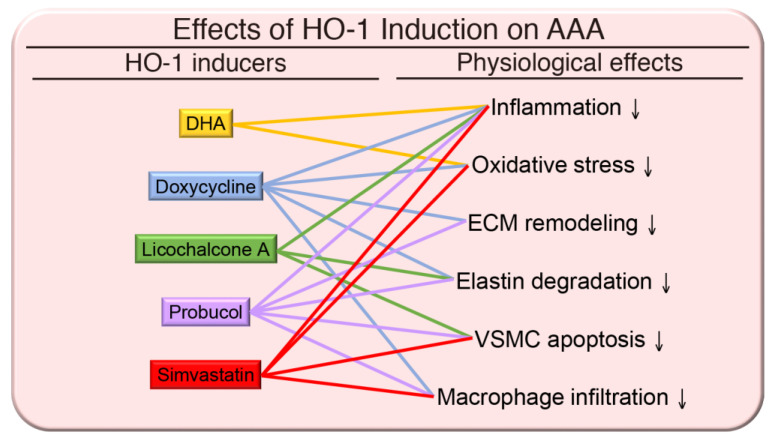
Effects of HO-1 induction on abdominal aortic aneurysm (AAA). The pathophysiology of AAA includes enhanced inflammation, oxidative stress, extracellular matrix (ECM) remodeling, elastin degradation, and vascular smooth muscle cell (VSMC) apoptosis in the media, and macrophage infiltration into the aneurysmal site. Upregulation of HO-1 by inducers including docosahexaenoic acid (DHA), doxycycline, licochalcone A, probucol, and simvastatin may alleviate some of the pathological outcomes of AAA.

On account of the aforementioned reasons, numerous studies attempted to pharmacologically inhibit inflammation, ROS generation, and ECM degradation to prevent aneurysm formation and/or progression through modulating HO-1 expression. Given that macrophages are a prominent cell type in AAA and that omega-3 polyunsaturated fatty acids (n-3 PUFA) have been suggested to attenuate adverse outcomes associated with inflammation, oxidative stress and disturbed antioxidant status, thus, it was hypothesized that n-3 PUFA and docosahexaenoic acid (DHA) might provide a therapeutic strategy for AAA [64]. Incubation of monocyte-derived macrophages obtained from male patients that had small AAA (3.0–4.5 cm diameter) with DHA-suppressed LPS-induced ROS and inflammatory cytokine levels. Importantly, DHA increased HO-1 mRNA levels and glutathione peroxidase activity in macrophages [64], indicating DHA is a promising target for further investigation in patients with AAA. MMP-9 expression in macrophages also has a potential role in AAA development. Doxycycline, a safe and commonly used antibiotic, inhibits MMP-9 expression and activity [65]. In an angiotensin II-induced AAA model, doxycycline markedly reduced the incidence and severity of AAA [66]. Doxycycline also had a preventive and therapeutic effect on AAA in a mouse elastase-induced AAA model, in part, interestingly, through upregulating HO-1 expression [67]. In a prospective, double-blind, randomized, placebo-controlled study, doxycycline significantly decreased the expansion rate of AAA [68]. A further prospective phase II multicenter study showed that prolonged administration of doxycycline (twice daily for 6 months) was safe and well tolerated in patients with small AAAs and was associated with a gradual reduction in plasma MMP-9 levels [69]. It appears that doxycycline also reduces vascular inflammation and selectively depletes neutrophils and cytotoxic T cells in the aortic wall of AAA patients [70]. Although not measured in these clinical studies, it is likely that doxycycline’s effects might be mediated through HO-1-dependent pathways. Licochalcone A, a compound extracted from the *Glycyrrhiza* species, has a strong potential to ameliorate angiotensin II-induced AAA via elevating HO-1 expression [71]. Probucol is a cholesterol-lowering drug and antioxidant and clinically used for the treatment of restenosis. Probucol induces HO-1 expression, inhibits VSMC proliferation and reduces restenosis [72]. A recent study demonstrated that probucol attenuates the development of AAA through HO-1 upregulation and protects against elastin degradation while facilitating elastin synthesis [73]. These HO-1 regulators and their effects on the pathophysiology of AAA are depicted in Figure 2.

Statins are a widely used cholesterol-lowering drug that also reduce the generation of ROS and proinflammatory cytokines [74]. The pleiotropic effects of statins seem to be advantageous in AAA disease through the promotion of HO-1 [62]. In a mouse elastase model, treatment with simvastatin suppressed the formation of experimental AAAs in both normal and hypercholesterolemic mice, independent of its lipid-lowering effects. Simvastatin preserved medial elastin and VSMC content, reduced MMPs, and increased MMP inhibitor tissue inhibitor of metalloproteinase 1 (TIMP-1) in the aortic wall in these mouse studies [75]. Corroborating with the results from this animal study, statins may attenuate AAA growth [76] and reduce the ROS level in the human AAA aortic wall via NF-κB signaling [77]. A more recent study also showed that statin therapy may reduce AAA progression, rupture, and lower rates of perioperative mortality following elective AAA repair [78]. Intriguingly, administration of statins significantly upregulated HO-1 expression in both endothelial cells (ECs) and VSMCs of AAAs in patients [79].

Taken together, numerous studies support the concept that HO-1 exhibits multiple protective functions in the vasculature. Therefore, activation of HO-1 expression clinically might be a promising therapeutic strategy for prevention or treatment of AAA.

## 3. HO-1 in Intracranial Aneurysm

Intracranial aneurysm is a cerebrovascular disorder also known as a brain aneurysm. Similar to atherosclerotic lesion, IA usually occurs at branch points along the intracranial arteries. However, in contrast to the luminal occlusion present in atherosclerosis, hemodynamic stress on the intracranial arterial wall between the two exiting branches weakens that region, resulting in IA formation [13,80]. The similarity in risk factors and pathological elements of IAs and AAAs suggests a shared pathophysiology and therefore HO-1 induction might also limit the severity of IAs by reducing these pathogenic cellular processes. Data from recent animal studies with activators of nuclear factor erythroid 2-related factor 2 (Nrf2), the major transcriptional activator of HO-1 [81], further emphasized a potential protective role of HO-1 against IA formation and progression. In a rat IA model with elastase treatment and ligation of unilateral common carotid artery, the Nrf2 agonist tert-butylhydroquinone (tBHQ) reduced incidence and severity of IAs. Nrf2 activation also prevented oxidative stress-induced switching of VSMCs from a contractile to a synthetic phenotype and apoptosis. Nrf2 activation also promoted the expression of antioxidant enzymes and VSMC-specific marker genes and decreased proinflammatory cytokine levels [82]. Dimethyl fumarate (DMF), which exhibits immunomodulatory properties, partly via activation of the Nrf2-mediated oxidative responses, also reduced the formation of IA in mouse models [83]. These studies indicate an essential protective role of HO-1 in IAs.

In addition to tBHQ and DMF, several HO-1 enhancing agents appear to be beneficial at least in IA animal models. Erythropoietin (EPO) attenuates pulmonary vascular remodeling through mobilizing endothelial progenitor cells (EPCs) and activating HO-1 [84]. In a rat IA model with ligation of carotid and renal arteries, EPO increased circulating EPCs, reduced the formation and progression of IAs, and reduced the expression of inducible nitric oxide synthase (iNOS) and MMPs [85]. Dipeptidyl peptidase-4 (DPP-4) inhibitors including sitagliptin have been shown to have beneficial effects in alleviating vascular remodeling and cardiac infarction by reducing ROS via the Nrf2/HO-1 pathway [86,87]. Consistent with this notion, the DPP-4 inhibitor anagliptin inhibited the accumulation of macrophages in IAs and prevented IA growth. Further, anagliptin reduced the production of proinflammatory cytokines monocyte chemotactic protein 1 (MCP-1), TNF-α, and IL-6. These results indicate that anagliptin prevents the growth of IAs via its anti-inflammatory effects on macrophages [88]. The suppressive functions of EPO and anagliptin in IAs may be attributed in part to HO-1 expression, although further studies are needed. Some evidence indicates that statins provide protection for AAAs in animal models and also in humans. However, the effects of statins on IAs are controversial. Some statins, such as atorvastatin [89], simvastatin [90], pitavastatin [91], and pravastatin [92] showed protective effects in animal IA models. In contrast, statins have been reported to promote the growth of experimentally-induced IAs in estrogen-deficient rats, although a low dose of pravastatin does reduce the incidence of IA [93], suggesting a possibility of differential effects by statins, particularly in older female patients with low estrogen levels. An additional study showed that in contrast to a positive correlation between statins and a reduction of IA formation in animal models, statins may have no significant beneficial effect on IA suppression in humans [94].

Endovascular coil embolization treatment for IAs has a high rate of recurrence [28]; as such, prevention of recurrence following embolization has become one of the essential issues of aneurysm intervention. Endothelialization at the aneurysmal neck and fundus is crucial in sealing off the aneurysm from the arterial circulation and stopping blood flow into the aneurysm [95]. Indeed, endothelialization has been observed in both clinical cases [96] and animal models [97]. Although the biological processes of postembolization healing and recurrence of the treated aneurysm are still far from fully understood, recruitment of ECs to the site of the aneurysmal neck and subsequent endothelialization are thought to be essential in ensuring adequate healing and preventing recanalization [98]. Evidence suggests that bone marrow derived-EPCs contribute to endothelialization of grafts and denuded arteries [99]. Interestingly, overexpression of HO-1 facilitates re-endothelialization of denuded vessels by promoting mobilization of EPCs while HO-1-deficiency hampers EPC mobilization and re-endothelialization [100]. Compared with wild type mice, bone marrow cells from HO-1 knockout mice generate fewer endothelial colony-forming cells [101]. Collectively, these findings implicate that HO-1 inducers may serve as a novel therapeutic option in postembolization management against aneurysm recurrence by enhancing endothelialization.

**Figure 3:**
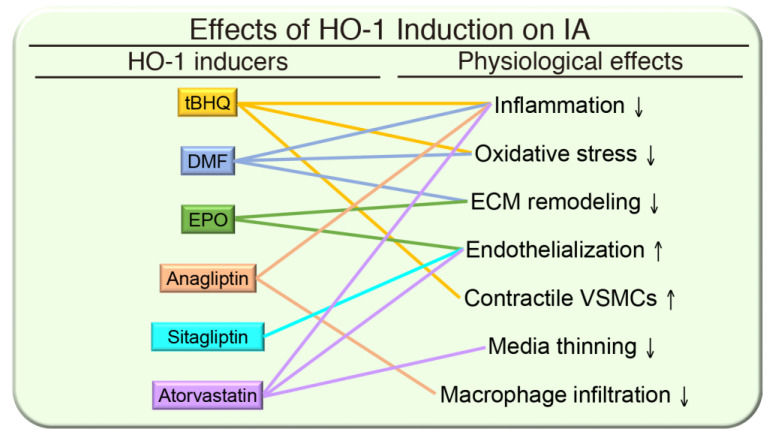
Effects of HO-1 induction on intracranial aneurysm (IA). The pathophysiology of IA includes enhanced inflammation, oxidative stress, extracellular matrix (ECM) remodeling, denudation of endothelium, phenotypic modulation of vascular smooth muscle cells (VSMCs) from a contractile to a synthetic phenotype, loss of VSMCs in the media, and macrophage infiltration into the aneurysmal site. Upregulation of HO-1 by inducers tert-butylhydroquinone (tBHQ), dimethyl fumarate (DMF), erythropoietin (EPO), anagliptin, sitagliptin, and atorvastatin attenuates development of IA and alleviates associated pathological effects.

DPP-4 inhibitors not only prevent IA growth and alleviate vascular remodeling via Nrf2/HO-1 pathway as discussed above, but the DPP-4 inhibitor sitagliptin facilitates EPC mobilization and endothelialization in aneurysmal necks, likely through Nrf2 signaling by promoting proliferation, migration, invasion, and angiogenic abilities of EPCs [102]. EPO not only reduces IA development [85], it also stimulates EPCs to endothelialize the aneurysmal neck after coil embolization by modulating vascular endothelial growth factor [103]. The role of statins in IA recanalization is less clear; both positive and negative results were reported from animal and clinical studies. Rosuvastatin was shown to enhance endothelialization of the coiled aneurysm neck via induction of EPCs in rats [104], whereas systemic simvastatin administration following platinum coil embolization of unruptured IAs did not improve aneurysm occlusion in rabbits [105]. In one human study, statins were associated with a lower rate of aneurysm recurrence following endovascular coiling of small- and medium-sized ruptured aneurysms [106]. Two other studies found a lack of association between statin use and angiographic and clinical outcomes after Pipeline embolization for IAs [107,108]. Taken together, enhancing HO-1 expression might protect against IA development, attenuate pathophysiological outcomes, and decrease recurrence after endovascular interventions (Figure 3).

Given that Pipeline embolization is often used on large aneurysms with wide necks it remains to be determined whether these medical devices used for intervention and/or characteristics of IAs affect the outcomes. Nonetheless, re-endothelialization provides an additional therapeutic opportunity for IAs following interventional procedures. Additional studies are needed to adequately evaluate the safety and efficacy of the potential targets for recurrence prevention after endovascular interventions. Further investigation and clinical trials would be required to determine the safety and efficacy of inducing agents to suppress or prevent human IA recurrence prevention.

## 4. HO-1 in Subarachnoid Hemorrhage

Following initial bleeding in SAH, blood accumulates within the subarachnoid space and penetrates into the brain parenchyma via paravascular pathways, including into areas remote from the initial bleeding site [109,110]. Blood-derived products such as hemoglobin and free heme have been implicated as key mediators of brain injury following SAH. Neurotoxicity of erythrocyte lysis, particularly hemoglobin, has been demonstrated in cortical cell culture assays [110,111,112]. Free heme, one major breakdown product of hemoglobin, can act as a potent cytotoxic prooxidant and proinflammatory molecule, causing cellular oxidative damage, cytotoxicity, and inflammation [113]. Neurotoxicity of hemin, the oxidized form of heme, has been reported in rat brain organotypic slices [114]. In primary cultures of rat cerebellar granule neurons treated with hemin, pretreatment of curcumin increased HO-1 expression and glutathione levels, attenuated ROS production and neuronal death, suggesting the protective effects of curcumin is likely mediated through HO-1 and glutathione [115]. In a rat model using lysed blood, whole blood, or oxyhemoglobin injection to mimic SAH, HO-1 was induced predominantly in microglia throughout the brain [116]. It was proposed that the microglial HO-1 response could be protective against hemoglobin-induced lipid peroxidation and vasospasm by increasing the clearance of heme, sequestrating iron, and by increasing the production of the antioxidant bilirubin [116]. In a murine model of SAH, expression of HO-1 in microglia was necessary to attenuate neuronal cell death, vasospasm, impaired cognitive function, and clearance of cerebral blood burden [117]. Therefore, it was concluded that microglial HO-1 and the generation of its reaction product CO are essential for effective elimination of blood and heme after SAH. A recent study showed that HO-1 is induced in microglia following blood exposure [118]. Importantly, neuronal cells are protected from cell death by microglia cell medium conditioned with blood [118], further supporting a critical role of microglial HO-1 induction in protecting against neuronal damage after SAH. Microglia are resident immune cells of the brain, and as such microglial HO-1 may exert its protective actions against SAH-induced neuronal injuries by modulating neuroinflammatory processes. These studies emphasize the importance of blood clearance in the injury response to SAH and a crucial protective function of microglial HO-1 upregulation in SAH.

Posthemorrhage administration of Nrf2 activator, sulforaphane, tBHQ, or DMF, enhances expression of both Nrf2 and HO-1, reduces oxidative stress, and ameliorates early brain damage, including brain edema, blood-brain barrier (BBB) impairment, cortical necrosis and apoptosis, motor deficits, and cognitive dysfunction following SAH in animal models [119,120,121]. Microglial-specific HO-1 overexpression in mice further confirmed the protective function of HO-1 in neuronal injuries following SAH [117]. Further supporting the critical importance of microglial HO-1, HO-1 deficiency in neurons and astrocytes did not influence vasospasm or neuronal apoptosis following SAH, while HO-1 deficiency in microglia caused aggravated neuronal apoptosis, hematoma volume, vasospasm, and cognitive deficits post SAH. Moreover, CO inhalation after SAH rescued the absence of microglial HO-1, demonstrating the necessity of microglial HO-1/CO in injury responses to SAH, at least for the acute and subchronic phases [117]. The neuroprotective action of HO-1 may also involve its modulatory effects on inflammatory responses, which have been clearly demonstrated in AAA [62,63]. HO-1 may modulate inflammatory response by its reaction products, such as CO and bilirubin, or its action of heme clearance. In organotypic rat brain slice cultures, hemin has been shown to induce neuronal death through enhancing IL-1α-related signaling pathways [114]. Further implicating the therapeutic potential of HO-1 activators, as with AAAs, patients with SAH are frequent carriers of long GT repeats (>36) in the HO-1 gene promoter, with decreased inducibility of HO-1 expression [122].

HO-1 inducers that are derived from natural sources, including quercetin, curcumin, and resveratrol, may have therapeutic application to immune-mediated diseases [40]. In animal models of SAH, many HO-1-enhancing agents are therapeutically beneficial, by reducing post-SAH mortality, improving functional outcomes, and attenuating SAH-induced learning deficits. Several natural products or their derivatives exhibit a beneficial effect in SAH. Recombinant human milk fat globule-EGF factor 8 (rhMFGE8) administration alleviates brain edema and neurological deficits by decreasing brain water content and improving neurological functions following SAH. rhMFGE8 attenuates oxidative stress and increases expression of extracellular signal-regulated kinase (ERK), Nrf2, and HO-1 while the HO inhibitor tin protoporphyrin (SnPP IX) abolished this effect, suggesting the protective effect might be mediated via the Nrf2-HO-1 pathway [123]. As such, rhMFGE8 may be a potential therapeutic target to ameliorate early brain injury for SAH patients. Oleanolic acid, a natural plant triterpenoid, has been shown to activate Nrf2/HO-1 signaling in kidney injury induced by cyclosporine and reduces renal pathology by providing beneficial effects on inflammation and oxidative stress [124]. Interestingly, it has been shown that prophylactic administration of oleanolic acid could prevented BBB disruption in an autoimmune encephalomyelitis mouse model [125]. Given that disruption of BBB is the main cause of vasogenic brain edema induced by SAH, it was proposed that oleanolic acid may play a neuroprotective role by protecting the integrity of BBB and reducing vasogenic cerebral edema after SAH. Indeed, oleanolic acid effectively alleviates SAH-induced vasogenic brain edema, reduces BBB permeability, and decreases SAH severity, by increasing HO-1 expression in SAH rats [126].

Apoptosis and inflammation contribute to early brain damage following SAH [127]. Mangiferin, a natural glucosyl xanthone found in both mango and papaya, has anti-inflammatory effect via upregulation of HO-1 in a mouse sepsis-induced lung injury model [128]. In a rat perforation model of SAH, post-SAH administration of mangiferin alleviated early brain injury and decreased mortality after SAH [127]. Mangiferin exerts neuroprotective effect by attenuating oxidative stress, reducing mitochondria-related apoptosis and inflammasome activation, and lessening NF-κB activation and production of proinflammatory cytokines via the Nrf2/HO-1 pathway [127]. In addition to protective effects on some neurological diseases, rhynchophylline has been reported to alleviate early brain injury following SAH, including attenuation of brain edema, neurological deficits, BBB disruption, hippocampal apoptosis, inflammation, and oxidative stress, at least in part via activation of HO-1 pathway [129]. Resveratrol, a plant-derived polyphenolic compound and a scavenger of a number of free radicals, increases expression of various antioxidant enzymes, including HO-1, and has antioxidant properties [130]. Resveratrol pretreatment attenuates cerebral ischemic injury by upregulating the expression of Nrf2 and HO-1 in rats [131]. Resveratrol has been shown to have antiapoptotic effects in an early brain injury model [132], implicating a potential protective role in SAH. Consistent with this observation, resveratrol reduces brain injury after SAH by inhibiting oxidative stress and endoplasmic reticulum stress [133].

**Figure 4:**
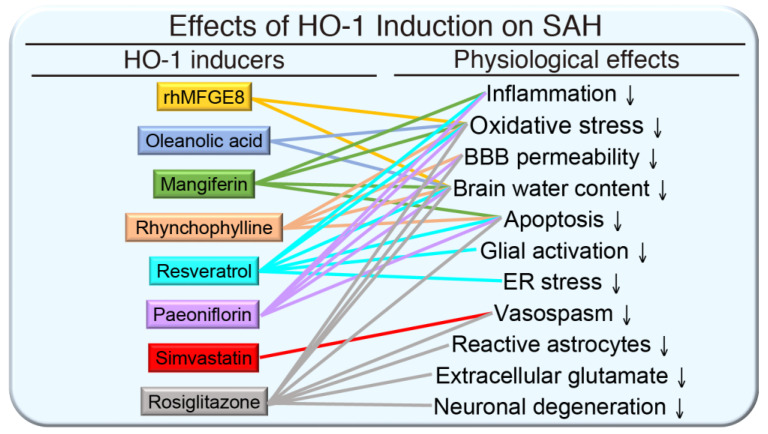
Effects of HO-1 induction on subarachnoid hemorrhage (SAH). The pathophysiology of SAH includes enhanced inflammation, oxidative stress, BBB permeability, brain water content, cellular apoptosis, glial activation, endoplasmic reticulum (ER) stress, vasospasm, reactive astrocytes, extracellular glutamate, and neuronal degeneration. Upregulation of HO-1 by inducers recombinant human milk fat globule-EGF factor 8 (rhMFGE8), oleanolic acid, mangiferin, rhynchophylline, resveratrol, paeoniflorin, simvastatin, and rosiglitazone attenuates SAH and alleviates some of the associated pathological effects.

In response to pathophysiological stress, heat shock proteins are upregulated and serve as the endogenous defense mechanisms. HO-1 is also known as heat shock protein 32. Interestingly, paeoniflorin, one of the major constituents of an herbal medicine derived from *Paeonia lactiflora*, is a heat shock protein-inducing compound [134]. Thus, paeoniflorin might protect against SAH-induced injuries via upregulating heat shock protein expression, including HO-1. Indeed, post-SAH administration of paeoniflorin leads to improvement of neuronal deficits and BBB integrity, reduction of neuronal apoptosis and pro-inflammatory cytokines, attenuation of microglia activation, decreases of oxidative stress, and enhancement of Nrf2/HO-1 pathway [135]. The diabetes drug rosiglitazone has been shown to induce HO-1 expression in human pulmonary alveolar epithelial cells and protects against lung inflammation [136]. Given the HO-1-inducing ability of rosiglitazone, it is not surprising that rosiglitazone attenuates early brain injury after experimental SAH [137,138], suggesting a possibility of drug repositioning. These HO-1-inducing agents support the concept that HO-1 induction is beneficial in protecting against SAH (Figure 4). A recent review also suggests a neuroprotective role of the Nrf2 pathway in SAH and its therapeutic potential [139].

Many HO-1-inducing agents have shown promise in improving the outcomes after SAH in animal models. However, human clinical studies are somewhat controversial, as some show promise while others do not. A preliminary clinical study showed that EPO therapy decreased the incidence of severe vasospasm, shortened the duration of impaired cerebral autoregulation, reduced delayed ischemia deficits, and resulted in more favorable outcome at discharge [140]. In a small case of seven patients with severe SAH, EPO treatment resulted in an increase in brain tissue oxygen tension significantly over baseline [141]. Nevertheless, another trial with 73 patients did not show conclusive beneficial effects of EPO in patients with SAH [142]. For statins, a pilot randomized clinical trial showed that the use of simvastatin as prophylaxis against delayed cerebral ischemia after aneurysmal SAH was safe and well-tolerated intervention [143]. In contrast, another trial did not detect any benefit in the use of simvastatin for long-term or short-term outcomes in patients with aneurysmal SAH [144]. A trial with pravastatin showed that acute treatment with pravastatin after aneurysmal SAH was safe and ameliorated cerebral vasospasm, improved cerebral autoregulation, and reduced vasospasm-related delayed ischemic deficits. Unfavorable outcomes at discharge was reduced primarily because of a reduction in overall mortality [145]. Another trial found that atorvastatin decreased CT and S100-assessed brain ischemia after SAH [146]. In contrast, a hospital-based case-control study in Japan suggested that there is an inverse relationship between the use of statins and IA rupture [147]. As such, it is not clear whether racial background is related to the effect of statins on IA progression. Therefore, the clinical effects of statins on IA remain uncertain. These clinical trials suggest that different statins may not be equivalent and large trials are needed to yield more definitive conclusion.

Another HO-1 inducer that could be clinically used is cilostazol, a potent type 3 phosphodiesterase inhibitor. Interestingly, the length polymorphism of GT-repeat in human HO-1 promoter determines the effect of cilostazol in VSMCs [148], further emphasizing the critical function of HO-1 inducibility in the therapeutic effect. Studies investigating the effects of cilostazol on aneurysmal SAH showed that cilostazol was safe and effective, and reduced the incidence of severe angiographic vasospasm, symptomatic vasospasm, new cerebral infarction, and poor outcomes [149,150,151]. The mechanisms underlying the therapeutic improvement by cilostazol are not yet fully elucidated. Besides vasospasm, one recent randomized clinical study with 55 aneurysmal SAH patients showed that cilostazol decreased the duration of spreading depolarization and spreading ischemia after SAH, suggesting repair of the neurovascular response to spreading depolarization by cilostazol [152]. These studies highlight the need for further large multicenter randomized clinical trials before cilostazol could be widely adopted in clinical practice. Altogether, animal and clinical studies have pointed to a therapeutic potential of HO-1 induction in SAH.

## 5. Conclusions

Aneurysm is a vascular disease with few obvious symptoms and is difficult to detect in the early stages. However, large aneurysms can cause serious illness and result in devastating consequences. Aneurysms are caused by specific genetic mutations, environmental factors, and enhanced inflammation and ROS levels which lead to medial VSMC loss and ECM degradation, resulting in progressive weakening of arterial structures. Rupture of aneurysms in the aorta has an extremely high mortality while rupture of IAs causes subarachnoid hemorrhage and stroke. To date, surgical intervention for severe aneurysms is the only option. Hence, preventing aneurysmal progression and/or regressing existing aneurysms is still an unmet medical need. Developing pharmacological means for aneurysmal diseases would also benefit patients and society from a socioeconomic aspect.

**Figure 5:**
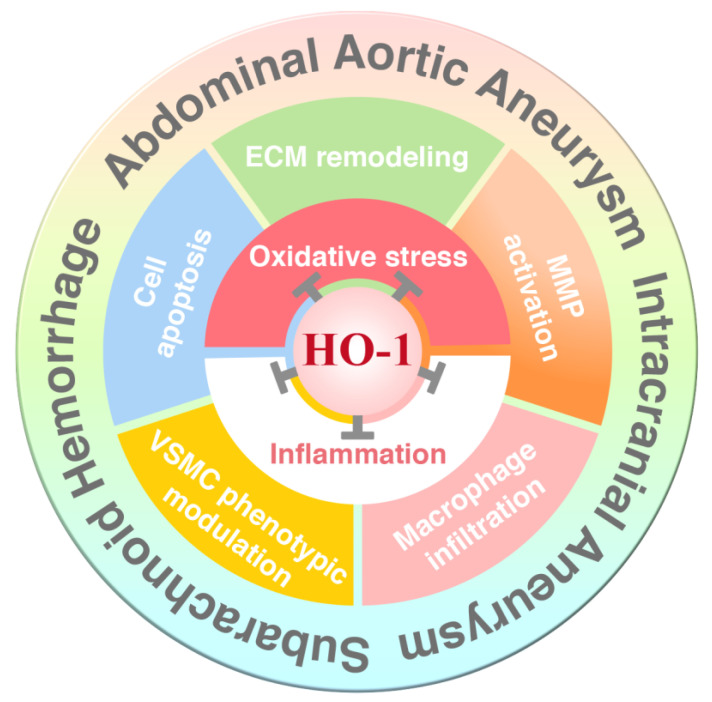
Schematic illustration of HO-1 in protecting against abdominal aortic aneurysm (AAA), intracranial aneurysm (IA), and subarachnoid hemorrhage (SAH). A number of pathophysiological effects of AAA, IA, and SAH, such as extracellular matrix (ECM) remodeling, matrix metalloproteinase (MMP) activation, macrophage infiltration, vascular smooth muscle cell (VSMC) phenotypic modulation, and cell apoptosis are linked to oxidative stress and inflammation. HO-1 possesses antioxidative and anti-inflammatory capacities, and thus upregulation of HO-1 reduces oxidative stress and inflammation, resulting in attenuation of the development and progression of these serious arterial diseases.

Numerous studies have identified the crucial protective role of HO-1 in multiple organs and cell types under pathological conditions. Given that HO-1 is expressed at low levels (except for a few select tissues) under basal physiological conditions, upregulation of HO-1 in response to injury and/or stress becomes an important therapeutic strategy. As such, many HO-1 activators, including natural products and their derivatives, statins, and current drugs that could be repositioned, have been identified and the protective function demonstrated in various animal models and in some small-scale clinical studies. Accumulating evidence suggests that a number of HO-1 inducers are effective in protecting against vascular-related diseases including AAA, IA, and SAH through inhibiting oxidative stress and inflammation (Figure 5).

Several clinical trials have been performed to investigate the therapeutic efficacy of HO-1 inducers in these disease settings. Although the results are mostly encouraging, conflicting findings suggest larger clinical trials may be needed to unequivocally establish clinical safety and therapeutic efficacy of a select HO-1 inducer. Given the intense investigation in this area, it is hopeful that the pharmacological use of HO-1 inducers in managing aneurysmal diseases could be realized in the near future.
